# 3-Cyclo­hexyl­sulfan­yl-2-(4-methyl­phen­yl)-5,7-dinitro-1*H*-indole

**DOI:** 10.1107/S1600536808027682

**Published:** 2008-09-06

**Authors:** P. Ramesh, A. Subbiahpandi, Ramaiyan Manikannan, S. Muthusubramanian, M. N. Ponnuswamy

**Affiliations:** a Department of Physics, Presidency College (Autonomous), Chennai 600 005, India; b Department of Organic Chemistry, School of Chemistry, Madurai Kamaraj University, Madurai 625 021, India; c Centre of Advanced Study in Crystallography and Biophysics, University of Madras, Guindy Campus, Chennai 600 025, India

## Abstract

In the title compound, C_21_H_21_N_3_O_4_S, the cyclo­hexane ring adopts a chair conformation. The nitro and methyl­phenyl groups are all coplanar with the indole ring system. Intra­molecular N—H⋯O and C—H⋯S hydrogen bonds generate *S*(6) ring motifs. The mol­ecules form *R*_2_^2^(20) centrosymmetric dimers *via* inter­molecular C—H⋯O hydrogen bonds. A short O⋯O contact [2.842 (2) Å] is observed in the dimer.

## Related literature

For related literature, see: Cordell (1981 ▶); Farhanullah *et al.* (2004 ▶). For details of hydrogen-bond motifs, see: Bernstein *et al.* (1995 ▶). For puckering parameters, see: Cremer & Pople (1975 ▶).
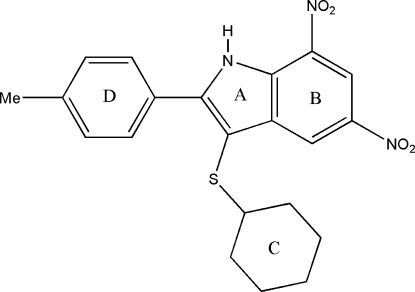


## Experimental

### 

#### Crystal data

C_21_H_21_N_3_O_4_S
*M* *_r_* = 411.47
Triclinic, 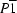

*a* = 6.1009 (3) Å
*b* = 8.5237 (4) Å
*c* = 19.1522 (10) Å
α = 83.551 (3)°
β = 84.184 (3)°
γ = 81.157 (2)°
*V* = 974.30 (8) Å^3^
*Z* = 2
Mo *K*α radiation
μ = 0.20 mm^−1^
*T* = 293 (2) K
0.30 × 0.20 × 0.16 mm

#### Data collection

Bruker Kappa APEXII area-detector diffractometer
Absorption correction: multi-scan (*SADABS*; Sheldrick, 2001 ▶) *T*_min_ = 0.953, *T*_max_ = 0.969
21439 measured reflections
4586 independent reflections
3424 reflections with *I* > 2σ(*I*)
*R*_int_ = 0.036

#### Refinement

*R*[*F* ^2^ > 2σ(*F* ^2^)] = 0.049
*wR*(*F* ^2^) = 0.170
*S* = 1.05
4586 reflections
267 parameters
H atoms treated by a mixture of independent and constrained refinement
Δρ_max_ = 0.54 e Å^−3^
Δρ_min_ = −0.31 e Å^−3^

### 

Data collection: *APEX2* (Bruker, 2004 ▶); cell refinement: *APEX2*; data reduction: *SAINT* (Bruker, 2004 ▶); program(s) used to solve structure: *SHELXS97* (Sheldrick, 2008 ▶); program(s) used to refine structure: *SHELXL97* (Sheldrick, 2008 ▶); molecular graphics: *ORTEP-3* (Farrugia, (1997 ▶)); software used to prepare material for publication: *SHELXL97* and *PLATON* (Spek, 2003 ▶).

## Supplementary Material

Crystal structure: contains datablocks global, I. DOI: 10.1107/S1600536808027682/ci2656sup1.cif

Structure factors: contains datablocks I. DOI: 10.1107/S1600536808027682/ci2656Isup2.hkl

Additional supplementary materials:  crystallographic information; 3D view; checkCIF report

## Figures and Tables

**Table 1:** Hydrogen-bond geometry (Å, °)

*D*—H⋯*A*	*D*—H	H⋯*A*	*D*⋯*A*	*D*—H⋯*A*
N1—H1⋯O1	0.82 (2)	2.30 (2)	2.755 (2)	115 (2)
C19—H19⋯S1	0.93	2.62	3.347 (2)	135
C22—H22⋯O2^i^	0.93	2.58	3.224 (3)	127

Symmetry code: (i) 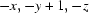.

**Fig. 1.**
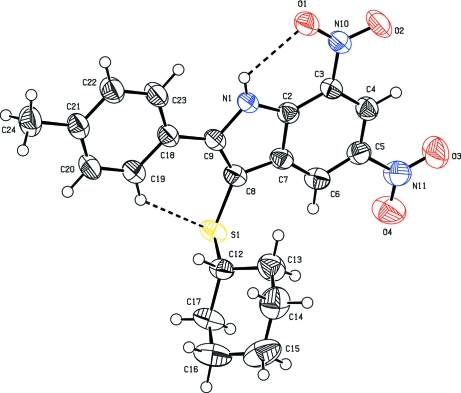
Molecular structure of the title compound, showing the atomic numbering and 50% probability displacement ellipsoids. Dashed lines indicate hydrogen bonds.

## supplementary crystallographic information

### Comment

Indole, being an integral part of many natural products of therapeutic importance, possesses potentially reactive sites for a variety of chemical reactions to generate molecular diversity (Farhanullah *et al.*, 2004). The spiro-indole ring system is a frequently encountered structural motif in many biologically important and pharmacologically relevant alkaloids, *e.g.* vincrinstine, vinblastine and spirotypostatins (Cordell, 1981). Against this background and to ascertain the detailed information on its molecular conformation, the structure determination of the title compound was carried out.

The indole ring system is planar and the two nitro groups are coplanar with it. The cyclohexane ring adopts a chair conformation, with puckering parameters (Cremer & Pople, 1975) q_2_ = 0.010 (3) Å, q_3_ = 0.574 (3) Å and φ = 51 (18)°. The methylphenyl group is also coplanar with the indole ring system [dihedral angle 1.98 (9)°]. Each of the intramolecular N1—H1···O1 and C19—H19···S1 hydrogen bonds generates an S(6) ring motif (Bernstein *et al.* 1995).

In the crystal structure,  molecules at (x, y, z) and (-x, 1-y, -z) are linked into a centrosymmetric *R*_2_^2^(20) dimer by C22—H22···O2 hydrogen bonds. Within the dimer, a short O1···O1 contact [2.842 (2) Å] is observed.

### Experimental

A solution of 2-(cyclohexylsulfanyl)-1-(4-methylphenyl)-1-ethanone- *N*-(2,4-dinitrophenyl)hydrazone (0.001 mol) in dimethylforamide (5 ml) was allowed to cool in an ice bath with stirring. To this stirred solution, phosphorus oxychloride (0.008 mol) was added dropwise and the mixture was subjected to microwave irritation for 30–60 sec under 40% power with a pulse rate of 15 s. The reaction was monitored by TLC and after completion of the reaction, the reaction mixture was poured onto the crushed ice. The solid was filtered and washed with plenty of water. The different compounds present in the mixture were separated by column chromatography using petroleum ether and ethyl acetate mixture as eluent. The title compound was recrystallized in dichloromethane in 10% yield.

### Refinement

The N-bound H atom was located in a difference map and refined freely. C-bound H atoms were positioned geometrically (C-H = 0.93–0.98 Å) and allowed to ride on their parent atoms, with *U*_iso_(H) = 1.5*U*_eq_(C) for methyl H and 1.2*U*_eq_(C) for other H atoms.

### Crystal data

**Table:**

C_21_H_21_N_3_O_4_S	*Z* = 2
*M**_r_* = 411.47	*F*(000) = 432
Triclinic, *P*1	*D*_x_ = 1.403 Mg m^−^^3^
Hall symbol: -P 1	Mo *K*α radiation, λ = 0.71073 Å
*a* = 6.1009 (3) Å	Cell parameters from 4582 reflections
*b* = 8.5237 (4) Å	θ = 1.1–27.9°
*c* = 19.1522 (10) Å	µ = 0.20 mm^−^^1^
α = 83.551 (3)°	*T* = 293 K
β = 84.184 (3)°	Block, colourless
γ = 81.157 (2)°	0.30 × 0.20 × 0.16 mm
*V* = 974.30 (8) Å^3^	

### Data collection

**Table:**

Bruker Kappa APEXII area-detector diffractometer	4586 independent reflections
Radiation source: fine-focus sealed tube	3424 reflections with *I* > 2σ(*I*)
graphite	*R*_int_ = 0.036
ω and φ scans	θ_max_ = 27.9°, θ_min_ = 1.1°
Absorption correction: multi-scan (SADABS; Sheldrick, 2001)	*h* = −7→8
*T*_min_ = 0.953, *T*_max_ = 0.969	*k* = −11→11
21439 measured reflections	*l* = −25→25

### Refinement

**Table:**

Refinement on *F*^2^	Primary atom site location: structure-invariant direct methods
Least-squares matrix: full	Secondary atom site location: difference Fourier map
*R*[*F*^2^ > 2σ(*F*^2^)] = 0.049	Hydrogen site location: inferred from neighbouring sites
*wR*(*F*^2^) = 0.170	H atoms treated by a mixture of independent and constrained refinement
*S* = 1.05	*w* = 1/[σ^2^(*F*_o_^2^) + (0.0969*P*)^2^ + 0.289*P*] where *P* = (*F*_o_^2^ + 2*F*_c_^2^)/3
4586 reflections	(Δ/σ)_max_ = 0.043
267 parameters	Δρ_max_ = 0.54 e Å^−^^3^
0 restraints	Δρ_min_ = −0.31 e Å^−^^3^

### Special details

**Table:**

Geometry. All e.s.d.'s (except the e.s.d. in the dihedral angle between two l.s. planes) are estimated using the full covariance matrix. The cell e.s.d.'s are taken into account individually in the estimation of e.s.d.'s in distances, angles and torsion angles; correlations between e.s.d.'s in cell parameters are only used when they are defined by crystal symmetry. An approximate (isotropic) treatment of cell e.s.d.'s is used for estimating e.s.d.'s involving l.s. planes.
Refinement. Refinement of *F*^2^ against ALL reflections. The weighted *R*-factor *wR* and goodness of fit *S* are based on *F*^2^, conventional *R*-factors *R* are based on *F*, with *F* set to zero for negative *F*^2^. The threshold expression of *F*^2^ > σ(*F*^2^) is used only for calculating *R*-factors(gt) *etc*. and is not relevant to the choice of reflections for refinement. *R*-factors based on *F*^2^ are statistically about twice as large as those based on *F*, and *R*- factors based on ALL data will be even larger.

### Fractional atomic coordinates and isotropic or equivalent isotropic displacement parameters (Å^2^)

**Table:**

	*x*	*y*	*z*	*U*_iso_*/*U*_eq_	
S1	0.37693 (9)	0.57116 (6)	0.32294 (3)	0.04681 (19)	
O1	−0.1393 (3)	0.6217 (2)	0.03507 (9)	0.0579 (4)	
O2	−0.4543 (3)	0.7672 (3)	0.05530 (10)	0.0796 (7)	
O3	−0.6221 (3)	1.0174 (3)	0.26759 (12)	0.0825 (6)	
O4	−0.3829 (4)	0.9921 (3)	0.34373 (11)	0.0856 (7)	
N1	0.1558 (3)	0.5458 (2)	0.13764 (10)	0.0390 (4)	
H1	0.157 (4)	0.513 (3)	0.0989 (13)	0.038 (6)*	
C2	−0.0106 (3)	0.6496 (2)	0.16537 (10)	0.0372 (4)	
C3	−0.2102 (3)	0.7282 (2)	0.14075 (10)	0.0394 (4)	
C4	−0.3508 (3)	0.8295 (2)	0.18166 (12)	0.0442 (5)	
H4	−0.4843	0.8820	0.1657	0.053*	
C5	−0.2909 (4)	0.8521 (2)	0.24679 (11)	0.0444 (5)	
C6	−0.0973 (4)	0.7772 (2)	0.27392 (11)	0.0444 (5)	
H6	−0.0619	0.7956	0.3181	0.053*	
C7	0.0435 (3)	0.6731 (2)	0.23291 (11)	0.0395 (4)	
C8	0.2509 (3)	0.5755 (2)	0.24487 (11)	0.0403 (4)	
C9	0.3180 (3)	0.4986 (2)	0.18474 (10)	0.0383 (4)	
N10	−0.2723 (3)	0.7050 (2)	0.07243 (10)	0.0481 (4)	
N11	−0.4432 (4)	0.9614 (2)	0.28929 (11)	0.0577 (5)	
C12	0.3142 (3)	0.3850 (2)	0.37195 (10)	0.0419 (4)	
H12	0.4020	0.2959	0.3489	0.050*	
C13	0.0732 (4)	0.3632 (3)	0.37755 (15)	0.0607 (6)	
H13A	0.0268	0.3586	0.3309	0.073*	
H13B	−0.0175	0.4531	0.3981	0.073*	
C14	0.0406 (5)	0.2098 (3)	0.42342 (17)	0.0743 (8)	
H14A	−0.1161	0.1982	0.4283	0.089*	
H14B	0.1219	0.1197	0.4007	0.089*	
C15	0.1201 (6)	0.2090 (4)	0.49543 (16)	0.0852 (10)	
H15A	0.0299	0.2933	0.5199	0.102*	
H15B	0.1024	0.1080	0.5227	0.102*	
C16	0.3607 (6)	0.2331 (4)	0.49010 (16)	0.0825 (9)	
H16A	0.4528	0.1429	0.4703	0.099*	
H16B	0.4052	0.2390	0.5368	0.099*	
C17	0.3957 (5)	0.3857 (3)	0.44377 (13)	0.0603 (6)	
H17A	0.3161	0.4768	0.4662	0.072*	
H17B	0.5528	0.3959	0.4385	0.072*	
C18	0.5160 (3)	0.3879 (2)	0.16483 (11)	0.0400 (4)	
C19	0.6844 (4)	0.3372 (3)	0.20963 (12)	0.0499 (5)	
H19	0.6706	0.3738	0.2540	0.060*	
C20	0.8705 (4)	0.2343 (3)	0.18971 (13)	0.0530 (5)	
H20	0.9801	0.2029	0.2209	0.064*	
C21	0.8985 (3)	0.1762 (3)	0.12438 (13)	0.0467 (5)	
C22	0.7331 (4)	0.2260 (3)	0.07958 (14)	0.0560 (6)	
H22	0.7480	0.1890	0.0352	0.067*	
C23	0.5454 (4)	0.3299 (3)	0.09918 (12)	0.0518 (5)	
H23	0.4367	0.3615	0.0677	0.062*	
C24	1.1020 (4)	0.0629 (3)	0.10306 (16)	0.0616 (6)	
H24A	1.0768	0.0150	0.0621	0.092*	
H24B	1.1321	−0.0188	0.1410	0.092*	
H24C	1.2269	0.1202	0.0924	0.092*	

### Atomic displacement parameters (Å^2^)

**Table:**

	*U*^11^	*U*^22^	*U*^33^	*U*^12^	*U*^13^	*U*^23^
S1	0.0558 (3)	0.0444 (3)	0.0442 (3)	−0.0110 (2)	−0.0198 (2)	−0.0025 (2)
O1	0.0530 (9)	0.0732 (11)	0.0447 (9)	0.0112 (8)	−0.0118 (7)	−0.0152 (8)
O2	0.0584 (11)	0.1142 (16)	0.0618 (11)	0.0323 (10)	−0.0311 (9)	−0.0277 (11)
O3	0.0664 (12)	0.0930 (15)	0.0803 (14)	0.0314 (11)	−0.0120 (10)	−0.0287 (11)
O4	0.1046 (16)	0.0878 (14)	0.0585 (12)	0.0318 (12)	−0.0201 (11)	−0.0321 (11)
N1	0.0373 (8)	0.0439 (9)	0.0351 (9)	−0.0011 (7)	−0.0091 (7)	−0.0024 (7)
C2	0.0382 (9)	0.0360 (9)	0.0368 (10)	−0.0057 (7)	−0.0047 (7)	0.0006 (7)
C3	0.0396 (10)	0.0408 (10)	0.0368 (10)	−0.0033 (8)	−0.0070 (8)	0.0009 (8)
C4	0.0414 (10)	0.0416 (10)	0.0474 (12)	−0.0011 (8)	−0.0062 (8)	0.0013 (9)
C5	0.0493 (11)	0.0386 (10)	0.0427 (11)	−0.0002 (8)	−0.0021 (9)	−0.0028 (8)
C6	0.0542 (12)	0.0401 (10)	0.0388 (11)	−0.0042 (9)	−0.0080 (9)	−0.0026 (8)
C7	0.0435 (10)	0.0369 (10)	0.0385 (10)	−0.0079 (8)	−0.0080 (8)	0.0020 (8)
C8	0.0425 (10)	0.0386 (10)	0.0406 (11)	−0.0074 (8)	−0.0103 (8)	0.0012 (8)
C9	0.0364 (9)	0.0395 (10)	0.0394 (10)	−0.0065 (7)	−0.0103 (7)	0.0017 (8)
N10	0.0442 (9)	0.0563 (11)	0.0421 (10)	0.0028 (8)	−0.0110 (7)	−0.0045 (8)
N11	0.0683 (13)	0.0500 (11)	0.0498 (12)	0.0095 (9)	−0.0056 (9)	−0.0080 (9)
C12	0.0511 (11)	0.0393 (10)	0.0353 (10)	−0.0009 (8)	−0.0099 (8)	−0.0050 (8)
C13	0.0592 (14)	0.0618 (15)	0.0637 (16)	−0.0165 (12)	−0.0139 (11)	0.0015 (12)
C14	0.0739 (18)	0.0625 (16)	0.086 (2)	−0.0213 (14)	0.0013 (15)	0.0030 (15)
C15	0.123 (3)	0.0659 (18)	0.0593 (18)	−0.0139 (18)	0.0167 (18)	0.0067 (14)
C16	0.117 (3)	0.0775 (19)	0.0515 (16)	−0.0137 (18)	−0.0255 (16)	0.0155 (14)
C17	0.0774 (17)	0.0627 (14)	0.0435 (13)	−0.0102 (12)	−0.0243 (11)	0.0011 (11)
C18	0.0365 (9)	0.0397 (10)	0.0435 (11)	−0.0053 (8)	−0.0088 (8)	0.0022 (8)
C19	0.0439 (11)	0.0604 (13)	0.0445 (12)	−0.0005 (10)	−0.0111 (9)	−0.0040 (10)
C20	0.0392 (11)	0.0626 (14)	0.0554 (13)	−0.0009 (9)	−0.0161 (9)	0.0039 (11)
C21	0.0387 (10)	0.0424 (11)	0.0575 (13)	−0.0035 (8)	−0.0095 (9)	0.0031 (9)
C22	0.0504 (12)	0.0610 (14)	0.0555 (14)	0.0068 (10)	−0.0132 (10)	−0.0142 (11)
C23	0.0463 (11)	0.0566 (13)	0.0514 (13)	0.0081 (9)	−0.0184 (9)	−0.0093 (10)
C24	0.0446 (12)	0.0570 (14)	0.0777 (18)	0.0057 (10)	−0.0055 (11)	−0.0003 (12)

### Geometric parameters (Å, °)

**Table:**

S1—C8	1.744 (2)	C13—H13B	0.97
S1—C12	1.823 (2)	C14—C15	1.507 (4)
O1—N10	1.223 (2)	C14—H14A	0.97
O2—N10	1.215 (2)	C14—H14B	0.97
O3—N11	1.216 (3)	C15—C16	1.505 (5)
O4—N11	1.208 (3)	C15—H15A	0.97
N1—C2	1.347 (2)	C15—H15B	0.97
N1—C9	1.390 (2)	C16—C17	1.521 (4)
N1—H1	0.82 (2)	C16—H16A	0.97
C2—C3	1.396 (3)	C16—H16B	0.97
C2—C7	1.409 (3)	C17—H17A	0.97
C3—C4	1.373 (3)	C17—H17B	0.97
C3—N10	1.441 (3)	C18—C23	1.387 (3)
C4—C5	1.377 (3)	C18—C19	1.392 (3)
C4—H4	0.93	C19—C20	1.373 (3)
C5—C6	1.375 (3)	C19—H19	0.93
C5—N11	1.464 (3)	C20—C21	1.382 (4)
C6—C7	1.388 (3)	C20—H20	0.93
C6—H6	0.93	C21—C22	1.377 (3)
C7—C8	1.427 (3)	C21—C24	1.502 (3)
C8—C9	1.384 (3)	C22—C23	1.383 (3)
C9—C18	1.460 (3)	C22—H22	0.93
C12—C13	1.502 (3)	C23—H23	0.93
C12—C17	1.510 (3)	C24—H24A	0.96
C12—H12	0.98	C24—H24B	0.96
C13—C14	1.519 (4)	C24—H24C	0.96
C13—H13A	0.97		
			
C8—S1—C12	103.22 (9)	C15—C14—H14A	109.3
C2—N1—C9	110.57 (17)	C13—C14—H14A	109.3
C2—N1—H1	124.1 (16)	C15—C14—H14B	109.3
C9—N1—H1	125.3 (16)	C13—C14—H14B	109.3
N1—C2—C3	133.05 (19)	H14A—C14—H14B	108.0
N1—C2—C7	107.39 (17)	C16—C15—C14	111.1 (2)
C3—C2—C7	119.55 (18)	C16—C15—H15A	109.4
C4—C3—C2	120.10 (19)	C14—C15—H15A	109.4
C4—C3—N10	118.89 (18)	C16—C15—H15B	109.4
C2—C3—N10	121.01 (18)	C14—C15—H15B	109.4
C3—C4—C5	118.86 (19)	H15A—C15—H15B	108.0
C3—C4—H4	120.6	C15—C16—C17	110.5 (2)
C5—C4—H4	120.6	C15—C16—H16A	109.5
C6—C5—C4	123.55 (19)	C17—C16—H16A	109.5
C6—C5—N11	118.6 (2)	C15—C16—H16B	109.5
C4—C5—N11	117.81 (19)	C17—C16—H16B	109.5
C5—C6—C7	117.57 (19)	H16A—C16—H16B	108.1
C5—C6—H6	121.2	C12—C17—C16	111.0 (2)
C7—C6—H6	121.2	C12—C17—H17A	109.4
C6—C7—C2	120.35 (18)	C16—C17—H17A	109.4
C6—C7—C8	132.23 (19)	C12—C17—H17B	109.4
C2—C7—C8	107.42 (17)	C16—C17—H17B	109.4
C9—C8—C7	106.84 (17)	H17A—C17—H17B	108.0
C9—C8—S1	131.38 (15)	C23—C18—C19	116.87 (19)
C7—C8—S1	121.76 (16)	C23—C18—C9	120.83 (18)
C8—C9—N1	107.76 (17)	C19—C18—C9	122.3 (2)
C8—C9—C18	132.49 (18)	C20—C19—C18	121.5 (2)
N1—C9—C18	119.74 (18)	C20—C19—H19	119.3
O2—N10—O1	123.43 (19)	C18—C19—H19	119.3
O2—N10—C3	118.61 (18)	C19—C20—C21	121.4 (2)
O1—N10—C3	117.96 (17)	C19—C20—H20	119.3
O4—N11—O3	123.6 (2)	C21—C20—H20	119.3
O4—N11—C5	118.0 (2)	C22—C21—C20	117.5 (2)
O3—N11—C5	118.3 (2)	C22—C21—C24	121.3 (2)
C13—C12—C17	111.4 (2)	C20—C21—C24	121.2 (2)
C13—C12—S1	114.70 (15)	C21—C22—C23	121.4 (2)
C17—C12—S1	105.15 (15)	C21—C22—H22	119.3
C13—C12—H12	108.5	C23—C22—H22	119.3
C17—C12—H12	108.5	C22—C23—C18	121.3 (2)
S1—C12—H12	108.5	C22—C23—H23	119.3
C12—C13—C14	109.6 (2)	C18—C23—H23	119.3
C12—C13—H13A	109.8	C21—C24—H24A	109.5
C14—C13—H13A	109.8	C21—C24—H24B	109.5
C12—C13—H13B	109.8	H24A—C24—H24B	109.5
C14—C13—H13B	109.8	C21—C24—H24C	109.5
H13A—C13—H13B	108.2	H24A—C24—H24C	109.5
C15—C14—C13	111.5 (2)	H24B—C24—H24C	109.5
			
C9—N1—C2—C3	−178.5 (2)	C2—C3—N10—O2	175.0 (2)
C9—N1—C2—C7	0.4 (2)	C4—C3—N10—O1	175.8 (2)
N1—C2—C3—C4	179.9 (2)	C2—C3—N10—O1	−4.1 (3)
C7—C2—C3—C4	1.1 (3)	C4—C3—N10—O1	175.8 (2)
N1—C2—C3—N10	−0.2 (3)	C2—C3—N10—O1	−4.1 (3)
C7—C2—C3—N10	−179.03 (18)	C6—C5—N11—O4	6.6 (3)
C2—C3—C4—C5	0.2 (3)	C4—C5—N11—O4	−173.8 (2)
N10—C3—C4—C5	−179.71 (19)	C6—C5—N11—O3	−174.2 (2)
C3—C4—C5—C6	−0.6 (3)	C4—C5—N11—O3	5.4 (3)
C3—C4—C5—N11	179.72 (19)	C8—S1—C12—C13	−50.48 (19)
C4—C5—C6—C7	−0.2 (3)	C8—S1—C12—C17	−173.23 (16)
N11—C5—C6—C7	179.44 (19)	C17—C12—C13—C14	−57.0 (3)
C5—C6—C7—C2	1.5 (3)	S1—C12—C13—C14	−176.27 (18)
C5—C6—C7—C8	−178.8 (2)	C12—C13—C14—C15	56.9 (3)
N1—C2—C7—C6	178.97 (18)	C13—C14—C15—C16	−56.9 (3)
C3—C2—C7—C6	−1.9 (3)	C14—C15—C16—C17	55.5 (4)
N1—C2—C7—C8	−0.8 (2)	C13—C12—C17—C16	57.0 (3)
C3—C2—C7—C8	178.26 (17)	S1—C12—C17—C16	−178.1 (2)
C6—C7—C8—C9	−178.8 (2)	C15—C16—C17—C12	−55.6 (3)
C2—C7—C8—C9	1.0 (2)	C8—C9—C18—C23	−178.5 (2)
C6—C7—C8—S1	−0.1 (3)	N1—C9—C18—C23	0.5 (3)
C2—C7—C8—S1	179.67 (14)	C8—C9—C18—C19	0.7 (4)
C12—S1—C8—C9	−79.3 (2)	N1—C9—C18—C19	179.66 (19)
C12—S1—C8—C7	102.35 (17)	C23—C18—C19—C20	−0.1 (3)
C7—C8—C9—N1	−0.7 (2)	C9—C18—C19—C20	−179.3 (2)
S1—C8—C9—N1	−179.26 (15)	C18—C19—C20—C21	−0.2 (4)
C7—C8—C9—C18	178.3 (2)	C19—C20—C21—C22	0.3 (4)
S1—C8—C9—C18	−0.2 (4)	C19—C20—C21—C24	−179.7 (2)
C2—N1—C9—C8	0.2 (2)	C20—C21—C22—C23	−0.2 (4)
C2—N1—C9—C18	−178.97 (17)	C24—C21—C22—C23	179.8 (2)
O1—O1—N10—O2	0.00 (4)	C21—C22—C23—C18	−0.1 (4)
O1—O1—N10—C3	0.00 (14)	C19—C18—C23—C22	0.2 (3)
C4—C3—N10—O2	−5.1 (3)	C9—C18—C23—C22	179.5 (2)

### Hydrogen-bond geometry (Å, °)

**Table:**

*D*—H···*A*	*D*—H	H···*A*	*D*···*A*	*D*—H···*A*
N1—H1···O1	0.82 (2)	2.30 (2)	2.755 (2)	115 (2)
C19—H19···S1	0.93	2.62	3.347 (2)	135
C22—H22···O2^i^	0.93	2.58	3.224 (3)	127

Symmetry codes:  (i) −*x*, −*y*+1, −*z*.
